# Sex Bias in Differentiated Thyroid Cancer

**DOI:** 10.3390/ijms222312992

**Published:** 2021-11-30

**Authors:** Valentine Suteau, Mathilde Munier, Claire Briet, Patrice Rodien

**Affiliations:** 1 Department of Endocrinology, Diabetology and Nutrition, University Hospital Angers, 49000 Angers, France; valentine.suteau@chu-angers.fr (V.S.); claire.briet@chu-angers.fr (C.B.); 2 MITOVASC Institute, University of Angers, 49100 Angers, France; Mathilde.munier@univ-angers.fr; 3 Centre de Référence des Maladies Rares de la Thyroïde et des Récepteurs Hormonaux, CHU Angers, 4 Rue Larrey, CEDEX 9, 49000 Angers, France

**Keywords:** thyroid cancer, sex bias, estrogens, reactive oxygen species (ROS), oncogenes

## Abstract

Differentiated thyroid cancers are more frequent in women than in men. These different frequencies may depend on differences in patient’s behavior and in thyroid investigations. However, an impact on sexual hormones is likely, although this has been insufficiently elucidated. Estrogens may increase the production of mutagenic molecules in the thyroid cell and favor the proliferation and invasion of tumoral cells by regulating both the thyrocyte enzymatic machinery and the inflammatory process associated with tumor growth. On the other hand, the worse prognosis of thyroid cancer associated with the male gender is poorly explained.

The thyroid diseases stand, together with diabetes and obesity, among the most frequent endocrine diseases. A female predominance is well recognized for most of these thyroid diseases, ranging from thyroid dysfunctions [1,2] to some form of congenital thyroid dysgenesis [3,4], or diffuse or focal nodular enlargement of the thyroid tissue, including thyroid cancer [1,2,5].

The higher frequency of autoimmune thyroid diseases chronic lymphocytic or Hashimoto thyroiditis, as well as Graves’ disease, in women will not be addressed here, as it is a trait shared with many other autoimmune diseases [6,7]. The mechanisms of this female overrepresentation are, nevertheless, not fully understood. One of these putative mechanisms, skewed X chromosome inactivation, is discussed already in this Special Issue [7].

We will rather focus on sex bias in differentiated thyroid cancer and the possible role of sex hormones in this disequilibrium. This review is not an extensive nor an exhaustive one. It should rather be seen as a survey of demonstrated or still hypothetic mechanisms at work concerning the sex disequilibrium in thyroid cancer frequency and prognosis.

## 1. Epidemiological Aspects

### 1.1. An Increase in Incidence of Thyroid Cancers

The incidence of differentiated thyroid cancer has increased in the last few decades, all around the world, and this has been explained in part by changes in clinical practice. Indeed, the set-up of a systematic screening in some countries has led to an epidemy of small cancers, overdiagnosis, and overtreatment, as this was not associated with a change in mortality, whereas the increase in more clinically relevant thyroid cancer was of a more limited amplitude [8,9,10,11,12]. Also, a more recent abatement was noticed [9,11]. Women are more likely than men to be concerned by this increase in thyroid cancer incidence [9,13,14,15]. Although rates vary between countries, the increase in papillary thyroid cancer rates was more pronounced in women than in male [13]. The progression of incidence seems to be dependent on tumor size, with a significant increase in incidence in women for microcarcinomas (5–10 mm) [9]. Moreover, the female/male ratio changes with the age or rather with the reproductive life since the difference of ratio increases from 15 years and decreases around 50–55 years [9].

### 1.2. Is There a Diagnosis Bias for Microcarcinomas?

It is interesting to compare the reported incidence of thyroid microcarcinomas, deduced from the number of cases related to surgical procedures, to autopsy series. In fact, the latter do not show a higher frequency in women [16,17], but rather a trend, in some studies, of higher numbers in males [17,18,19,20]. Assessing the frequency of incidentally discovered microcarcinomas during a thyroidectomy gives a more precise indication of the respective frequencies of microcarcinomas in males and females which are close [9], rather than the incidence, extracted from registries, which may be biased by surgical practice (total or partial thyroidectomy) [21] and thyroid surgery volume [22,23,24], as well as patients behaviors such as frequent versus rare seek for medical care and easy access to investigations [24,25,26]. The picture is further complicated by gender specific behaviors leading to later diagnosis and larger tumors in men. Looking at the indications for thyroid ultrasound, eventually leading to the diagnosis of a thyroid carcinoma, it appears that the initial symptoms or the cause of medical investigations are more frequently unknown or unrelated to thyroid disease in women [27]. In other words, women are more likely to have thyroid investigations than men, whatever the reason why they see a doctor, including regular visits to the gynecologist [27,28,29,30,31]. Regional variability in cancer incidence is likely to reflect differences in practices rather than exposure to environmental factors [9]. In fact, even regional or nationwide population screening programs, expected to abrogate the gender differences in behavior, can result in a higher screening rate in women than in men and a subsequent higher incidence in females, which could be misinterpreted as an indication of unequal risks of microcarcinomas. South Korea screening program included more women than men, up to 1.9-fold more [8,32], which led to five times more diagnosis of thyroid cancer leaving us with a female:male ratio of three when trying to correct for the higher screening rate in women [32].

In addition, the extent of surgery, the age of the patient at surgery (rather than the age of patients with microcarcinomas), and the methodology used to identify microcarcinomas (thickness of thyroid slices, systematic examination of all slices, random sampling or examination only of slices with suspected lesions, …) can strongly impact the results of such studies [17]. It is expected that methodological issue would have the same consequences in male and female populations, except if the size of lesions differs between the two sexes.

The same frequency of microcarcinomas in autopsies series can be interpreted as an equal propensity of men and women to develop a microcarcinoma and a higher proliferation and growth of tumors in women. The frequency of thyroid carcinoma has been reported to be similar in boys and girls, with a difference becoming obvious at pubertal age [11,33,34]. The sex hormones likely promote growth of a preexisting tumor, whereas the appearance of the neoplasm likely reflects exposure to environmental factors and molecular injury, putatively on a genetic predisposing background. In fact, the female to male ratio in radiation induced thyroid cancers is lower, or even close to 1, than in non-radiation-induced cancers. This is especially true for children diagnosed with a thyroid cancer [35,36,37].

### 1.3. Genetic Forms of Thyroid Cancer

Interestingly, the higher risk of thyroid cancer in women is observed with respect to some, but not all, syndrome of familial non medullary thyroid cancers. A higher risk for women for having a thyroid cancer is known in families affected by familial adenomatous polyposis syndrome (FAP), which is due to mutations in the APC gene. Interestingly, thyroid cancer in this case is a peculiar variant, namely the cribriform-morular variant. The women to men ratio is higher than 60/1 [38]. Depending on the studies, the number of cases and the way the results are expressed, thyroid carcinoma also have a higher frequency in women affected by PTEN (phosphatase and TENsin homologue) Hamartoma tumor syndrome, encompassing the Cowden disease, Bannayan-Riley-Ruvalcaba syndrome, PTEN-related Proteus syndrome, and Proteus-like syndrome [39].

However, despite a higher number of cases in women, it appears that standardized incidence rates are much higher for men than for women harboring a mutation in the PTEN gene. This is also true for mutations in SDHB/D (succinate dehydrogenase B and D subunits) genes and KLLN (or Killin) promoter alterations [39], which lead to Cowden-like syndromes. This may be due to the low male incidence in the general population, amplifying in men the apparent effect of these genetic alterations and bringing a very high risk in both males and females.

The analysis of the gender-related risk of thyroid cancer is difficult for the DICER1 gene, coding for an endoribonuclease. Indeed, the very high prevalence and early occurrence of multinodular goiter, in this syndrome, frequently leads to thyroidectomy, which may precede the appearance of a thyroid cancer, thus minimizing its frequency. However, when pooling the thyroidectomies for multinodular goiters and thyroid cancers, a clear female predominance is observed in carriers of pathogenic DICER mutations. It is proposed that a male patient with a thyroid cancer or even a multinodular goiter, especially early in life, should be proposed a search for DICER syndrome [40], which illustrates the combining effect of the gender bias and of the genetic tumor predisposition. Another study starting from search for DICER1 variants in the population found the same number of thyroid cancers in males and females (one for each sex) among carriers of pathogenic variants [41].

Familial non medullary thyroid cancer, excluding thyroid cancer associated with known tumoral syndromes such as those already mentioned, also shows a female predominance, although variable according to the definition of familial cases (either two or at least three cases in the family) and the number of cases, with the risk of biased statistics when case numbers are low [42,43].

In summary, thyroid microcarcinomas have a similar incidence, when appropriately assessed, in women and men, whereas clinically significant thyroid carcinomas are more frequent in women.

### 1.4. Factors Associated with Reproductive Life

Several studies have analyzed the links between reproductive factors in women and the incidence of thyroid cancer.

Especially by working on populations with very high incidence of thyroid cancers such as Melanesian women in New Caledonia, French Polynesia, or in Hawai, it seemed that there might be a link with number of pregnancies, age at menarche, natural or artificial menopause, and hormonal contraceptive use. This can be seen as an indirect indication of the role of estrogens. However, the picture diverges among the different studies, either reflecting different population selections or in some case the interplay between genetic background, environmental factors (radiations in the case of French Polynesia), and cultural issues such as number of pregnancies, age at first or last pregnancy [44,45,46,47], etc. The associations were, however, either inconstant or weak [46,48,49] and sometimes significant only in women aged less than 45 [46,47,48].

The use of oral contraceptives was shown to be protective in a large population-based case control study, which may appear to contradict the higher risk associated with number of pregnancies in Polynesia [44] and in Europe [50] if one only considers the role of estrogens. However, preventing a pregnancy may be protective from thyroid cancer if the gestation associated risk includes hCG (human chorionic gonadotropin) stimulation and immunosuppression. In agreement with the role of oral contraceptives, a late age at first pregnancy appeared to be protective [51].

## 2. What Are the Possible Explanations for the Female Predominance?

### 2.1. The Female Trend to Thyroid Overgrowth

The prevalence of goiter and nodules is four to five-fold as high as the one in males [1,2,5]. The role of sex hormones in this higher frequency of goiters can be deduced by the very frequent enlargement of the thyroid gland during pregnancy, which points to the role of estrogens in proliferation of thyrocytes. However, the gestational increase in the thyroid size may be due also to stimulation by hCG, endowed with a TSH-like activity due the structural proximity of the two glycoproteic hormones, which is responsible for the early pregnancy increase in thyroid hormones [52].

### 2.2. Relation with Reproductive Life

The impact of pregnancy on the growth and aggressiveness of thyroid cancer has been debated. Although the overall outcome, in terms of mortality, of thyroid cancer either discovered during or preexisting to pregnancy is still very good [53,54,55], there are some indications that the risk of recurrence or persisting disease may be increased by pregnancy [53,56,57]. Non-operated micropapillary carcinomas showed also a trend to progression in size during gestation [58], although with no impact on outcome. Interestingly, the risk of progression during gestation was similar to the risk of the non-pregnant population of the same age [58,59].

The limited higher risk of progression may be related to several factors. A higher estrogenic stimulation may further enhance the female risk of thyroid cancer. The stimulation by the TSH-like activity of hCG also probably contributes to the progression. Finally, the transient immune suppression observed during gestation may favor the thyroid cancer growth. A change in the expression of estrogen receptors, especially ERα, has been proposed. However, there was no consistent trend between different studies [56,57,58].

More strikingly, epidemiological studies have shown that males and females have comparable thyroid volume during childhood, whereas a higher volume in females becomes the rule at the age of puberty, with a later attenuation of differences in gross volume due to the relation to body surface [4]. Again, the frequency of nodules is also higher in women than in men, and the difference persists along adult life [1,2,5].

Animal models have further stressed the higher likelihood for females of developing thyroid tumors in female [60,61,62]. The influence of gender was nicely illustrated in a murine model of predisposition to thyroid tumors and ultimately thyroid cancers. This allowed for the demonstration of the impact of sexual hormones, that could reverse the sex bias. It also gave some indication on the tumorigenic pathways affected by the estrogens [61,63].

### 2.3. Is the Thyroid Stimulation by Thyrotropin (TSH) Different in Women and Men?

The TSH normal range is roughly the same in males and females. However, variations in circulating TSH have been reported during the menstrual cycle by several authors [64,65]. It is known that estrogens increase the concentration of circulating thyroxin binding globulin (TBG), the specific high affinity binding protein for thyroid hormones. This results in a decrease in free circulating thyroxin and an increase in TSH as a response. It is particularly known for hypothyroid women treated by thyroid hormones who have to decrease their daily dose of thyroxin to avoid overtreatment upon menopause or when they stop an estrogenic treatment [66]. However, the reset of the thyrotropic axis results in a limited change in women with a normal thyroid function [66]. It has been shown that the mean TSH and 2–2.5th and 97–97.5th percentiles are higher in females than in men [67,68,69,70]. The contribution of oral contraceptives to this difference should not be overlooked [69]. It is conceivable that a continuous though extremely limited higher stimulation by TSH, participates both to higher thyroid volume in females after puberty, and higher frequency of clinically significant thyroid cancer. In fact, several studies have reported higher frequency of thyroid cancers with a higher TSH concentration, even in the normal range [71]. The nature of the relationship between TSH and thyroid cancer is not simple. As already mentioned, a higher TSH might contribute to thyrocyte proliferation. However, recent studies have indicated that genetic traits associated with TSH value have an inverse correlation with the probability of a thyroid cancer. In other words, the lower the predicted TSH, the higher the risk of thyroid cancer [72,73].

### 2.4. Is the Thyroid, Highly Mutagenic Cellular Milieu, Impacted by the Sex Hormones?

The thyroid cell is a mutagenic environment due to the mandatory high production of reactive oxygen species (ROS) used for thyroid hormone synthesis. This production of ROS is based on the apical machinery including NADPH oxydases [74,75,76]. Due to a strong polarization of the cells, building a tight apical border of the thyroid follicles, the ROS are largely confined to the follicular lumen [76,77]. However, there is also an intracellular production due to NOX4 [78]. It has been shown, that the ROS production system is involved, and necessary, for chromosomal rearrangement after irradiation of thyroid cells in vitro [79,80]. The highly mutagenic thyroid environment has been also demonstrated in vivo in an animal model, where the frequency of mutations of a reporter gene was much higher in the thyroid than in other organs [81]. This oxidase system has a higher expression in females and has been shown to be upregulated by estrogens [82,83,84,85]. No sex difference was specifically reported in the number of mutations in the nice model used by Maier et al. [81], studying males and females.

Very strong antioxidant systems are also present in the thyroid cell, which explains that the thyroid cells survive better than other cells after radiation or exposure to ROS and even proliferate if the DNA damages leads to oncogene activation or inactivation of tumor suppressor genes [86,87]. A decrease in some components of the antioxidant system, namely glutathione peroxidase and thioredoxin reductase, has been shown in thyroid cancer (albeit unfortunately without precision on the proportion of women in the population studied) [88]. In an animal model, the female higher ROS production in thyrocyte, was associated with a lower scavenging capacity [85]. The female thyrocyte may thus be more prone to self-administered DNA damages than the male one.

### 2.5. Estrogen Receptors Are Present in the Thyroid

The nuclear estrogen receptors (ER), α and β, as well as membrane GPCR for estrogen, GPER/GPR30, have been found to be expressed in thyroid, especially in thyroid carcinoma [89,90,91,92,93,94]. ERα has a higher expression in thyroid cancers compared to normal surrounding thyroid parenchyma, or to benign nodules [95]. ERβ has been found also, usually with a lower expression [93,96]. In addition, the progesterone receptor has been described in some studies, as well as the androgen receptor. The techniques used to identify and quantify the different receptors have varied with time, which can explain the differences, sometimes contradictions, between studies.

The cribriform-morular variant, mostly affecting women, has been shown to harbor a high expression of ERα and β, together with progesterone receptor, and more scarce androgen receptor positivity [97].

In an exploratory study on G Protein-coupled receptors expression in thyroid cancer, we did not find a differential expression of GPER/GPR30 between tumoral and surrounding parenchyma, nor did we find any sex difference in the GPCR profile of tumors [98].

### 2.6. What Role Do the Estrogens Play in the Appearance, Growth, Evolution of Thyroid Cancers?

Estrogens are obviously circulating at higher concentration in women than in men. However, the presence of aromatase has been demonstrated in the thyroid tissue, together with the ability to convert testosterone in estradiol [99]. In addition, the 17 beta hydroxysteroid dehydrogenase type 1, converting estrone to estradiol, that is a more potent estrogen, has been found also [94]. This allows the production of estradiol from adrenal androgens, via aromatization followed by reduction.

The presence of estrogens leads to estradiol-DNA adducts that have been found increased in patients with thyroid cancer. These estradiol-DNA adducts may favor DNA damages, although the role of this mechanism is debated [100,101].

Along with the description of the presence of estrogen receptors in thyroid cancers, a role in proliferation has been shown by many groups. Several studies have shown that treatment by estrogen or agonists of the ERs can enhance, whereas antagonists decrease, the proliferation of the malignant thyroid cells. This was shown both with permanent thyroid cell lines, or with primary cell lines, either of human or animal origin [93,102,103,104,105,106,107]. ERα stimulates proliferation, in part via the activation of ERK1 (extracellular regulated Kinase) and 2 [105], whereas ERβ decreases it [108].

### 2.7. The Stem Cells or Cancer Stem-Like Cells Hypothesis

The presence of stem cells or cancer stem cells in the thyroid gland and thyroid cancers as well as in other cancers has been debated, depending on the way such cells were identified [109,110]. One of the characteristics of these cells is their ability to grow as organoids in vitro and, contrasting with the limited capacity of thyroid cells to divide, their proliferative properties, providing explanations for the mutations found in thyroid cancers [109,110]. The thyroid cells identified as stem cells have been shown to be responsive to estrogens, especially in the organoid/spheroid model. A higher expression of estrogen receptors has been found in progenitors than in differentiated thyrocytes, and estradiol inhibited the differentiation induced by TSH, in vitro [111]. Estrogens were shown to increase the proliferation of such cells [111,112,113].

### 2.8. Interactions between Steroid Hormones and Oncogenic Pathways

Some tumor predisposing syndromes offer nice illustrations of the impact of estrogens on thyroid tumorigenesis and or tumor growth. The thyroid cancer associated with familial adenomatous polyposis (FAP) syndrome is frequently a variant form, namely the cribriform morula carcinoma, and affects almost exclusively women [38,98]. The tumors have a strong expression of estrogen receptors and a lighter one of androgen receptors [98]. It is also characterized by a strong positivity of β-catenin in the nuclei [98]. It is known that the nuclear accumulation of β-catenin participates to the proliferation signaling cascade and it has been shown that ERs and β-catenin can interact to potentiate their respective actions [114]. Genetically modified animal models can also bring some indications on the role of estrogens. A mice model of PTEN-loss associated thyroid cancer has been produced, showing a female higher frequency of the disease. This was related to a lower expression of CDKN1B (p27) in females [61]. It had been already shown that the PTEN loss, and subsequent PI3K/AKT-dependent proliferation in mice, was associated with goiter development and a higher proliferation in females [63]. The in vivo difference in proliferation of thyrocytes between males and females could be abolished by female castration or estrogenic treatment of males [61]. The treatment of PTEN-less thyroid cancer cell lines decreased the expression of p27, and the double genetic loss of PTEN and p27, abolished the sex difference in frequency of thyroid cancer [61], allowing for the identification of p27 as a mediator of estrogen effects of thyroid cancer.

Beside the models of familial diseases, murine models for sporadic thyroid cancer have also been produced. Mice with a transgenic thyroid expression of RET fusion genes develop papillary-type thyroid carcinoma with no apparent sex bias [115,116] and transgenic thyroid expression of mutant BRAF leads to the development of thyroid cancer, with dedifferentiation in some cases [117,118,119]. Interestingly, whereas no sex bias in the occurrence of cancer was described in this later model, severe invasiveness and dedifferentiation were more frequent in males in one study [119]. This may be due to the mouse strain, the model of transgenesis and/or to a much higher TSH observed in male early in life [119]. This bias was not mentioned in another study, despite the confirmation of the TSH-dependency of cancer appearance [118].

A post-natal thyroid expression of mutated BRAF (mimicking a somatic post-natal event), associated with a PTEN defect did not show any sex bias in the occurrence of thyroid cancer [120], nor in a multistep model of carcinogenesis and dedifferentiation by loss of p53 [121]. It has to be reminded that some animal models imply the treatment of animals by short courses of tamoxifen for the genetic modification to happen, which can have sustained effects, even after only one neonatal injection [122]. Whether this can lead to, modulate, or mask a sex bias in the occurrence or severity of the thyroid carcinoma, is not known. Also, in early studies the forced pre or postnatal expression of oncogenes is frequently of higher magnitude than the spontaneous one.

### 2.9. Impact of Estrogens on the Thyrocyte Response to Stress

Autophagy is a mechanism by which cells eliminate some toxic products and recycle others to sustain vital function. It is induced in condition of hypoxia, and Hif-1 (hypoxia induced factor) has been shown to participate to this activation [123], as well as during starvation. It is associated with higher cell survival, less apoptosis, and could contribute to thyroid cancer cell proliferation. Mice in which autophagy is genetically defective in thyroid cells, by knocking out the *ATG5* gene, have an increased rate of thyrocyte apoptotic death associated with DNA damages caused by increased ROS production. This ROS induced DNA damage is higher in male mice [124]. The same authors further investigated the regulation of autophagy in mice, concentrating on male mice, and showed that TSH enhanced this process [125]. Whether this would be observed to the same extent in female mice is not known. Fan et al. [126], showed that estradiol could increase autophagy in thyroid cancer cell lines where ERα was overexpressed to mimic the spontaneous overexpression observed in papillary cancer, relatively to surrounding normal tissue. This action of estrogen was associated with the generation of ROS and activation of ERK1/2. In the same model the pharmacological or genetic inhibition of autophagy lowered the cell proliferation and induced apoptosis. More recently, the chaperone mediated autophagy signaling cascade was shown to be inhibited by tamoxifen or ERα siRNA in thyroid papillary cancer cell lines [127]. The ERα action on chaperone mediated autophagy was relayed, again, by ROS generation and ERK1/2.

A defect in Caveolin-1 has been associated with higher thyrocytes proliferation. This may be a compensation for a higher cell lethality, or for a real deregulation of the thyrocyte functioning. Indeed, in the absence of caveolin, the Thyroxisome (the machinery for thyroglobulin iodination, including the H_2_O_2_ production) is mislocalized and accumulates inside the cell, rather than at the apical pole [77]. This may participate to thyroid injury in Hashimoto’s thyroiditis [77,128,129]. A decreased Caveolin-1 expression is induced by the activation of the thyrotropin receptor and contributes to the increase of thyrocyte proliferation [130]. Also, a decrease in Caveolin-1 has been described in follicular thyroid cancer when compared to healthy thyroid tissue, but not in papillary carcinomas [131,132]. The Caveolin-1 has been involved in the increased autophagy and decreased apoptosis induced by estrogen treatment in breast cancer cells [133,134,135]. As we did not find indications of regulation of caveolin by estrogens in the thyrocyte, its possible contribution to the sex bias in appearance and/or progression of thyroid cancer (via increased autophagy and decreased apoptosis) is not known. Considering the higher production of ROS in female thyrocytes, the same or close frequency of radiation induced carcinomas [36,37], which may be seen as initial lesions, more data are needed concerning a putative sex bias in the anti ROS system and in the thyrocyte, as it is known that the balance between oxidant and anti-oxidant system is more favorable in women [85,88].

### 2.10. The Cancerous Thyrocyte Environment

The proliferation of thyrocytes is crucial for the development of thyroid cancer. However, the development of an invasive tumor requires also the appearance of new properties such as migration, and invasiveness, and capacity to modulate the extracellular milieu by triggering or silencing immune and inflammatory reactions. Beside its role in proliferation, ERα plays a role in cell migration and invasion, as shown by the use of tamoxifen as antagonist [102]. Estrogens have been shown to enhance also the secretion of VEGF (vascular endothelial growth factor) by the thyroid cells [82,136], thus modulating the vascular environment and allowing for further growth of the tumor [136,137,138].

A lower expression of ERα is associated with a loss of adhesion, a mark of metastasis potential [139]. Bisphenol A, an estrogen-like endocrine disruptor stimulates thyrocyte proliferation [140], as well as epithelial mesenchymal transition [141].

Due to a high and fast centrifuge proliferation of cancer cells, hypoxic areas usually appear in growing tumors. This leads to the secretion of Hif-1α which stimulates a pro-inflammatory response and, together with ERα, stimulates invasiveness [142]. Several studies have assessed the expression and the role of different chemokines and chemokines receptors, in thyroid cancer cell lines or thyroid tumors. In contrast with results in some other cancers [143] and regulation by estrogen of some chemokine receptors [144,145,146] interacting with ERα in positive feedback, and despite the expected sex biased regulation of inflammation and chemokines [147,148,149], there is no indication of sex difference in the chemokine or chemokine receptor profile in thyroid cancer [150,151,152,153,154,155,156]. Again, we did not find any sex difference in the expression profile of chemokine receptors in our study of GPCR expression in thyroid cancer [98].

## 3. Why a Worse Prognosis in Men?

It is recognized, although debated, that there is an increased risk of poor outcome in men with thyroid cancer [157,158,159,160]. This over-risk of poor outcome has been debated due to the higher age and later diagnosis in men, together with a higher stage upon diagnosis, reflecting the different behavior and access to medical care which may bias this prognosis factor. In fact, the male sex really appears as an independent prognosis factor limited to patients harboring a BRAF mutation, which is by itself a prognostic factor [161]. The same is true for the age as a prognostic factor [162] although discussed as putatively less important than pTERT (telomerase reverse transcriptase) genetic alterations, which would deserve to be explored also, regarding the male gender were associated with poorer prognosis. It should be stressed that the frequency of BRAF mutation does not differ between men and women, in contrast to what is observed between younger and older patients and to the apparent higher frequency, 38.4%, of TERT promoter mutations in men (versus 22.8% in women) in a meta-analysis including eight studies [163]. If the higher frequency of thyroid cancer in women than in men is linked to the role of estrogens in proliferation, angiogenesis, and invasiveness, the higher aggressiveness in men leading to higher recurrence rate, and higher mortality is not easily explained. Similarly to the sex bias in frequency of thyroid carcinoma, the better prognosis in women (or worse prognosis in men) is demonstrated only for carcinoma over 10 mm in size [160,164].

Although the androgen receptor expression in normal thyroid tissue is higher in men than in women, a decrease has been shown in tumoral tissue compared to surrounding tissue, and the sex difference is no more significant [165]. The androgen receptor expression was independently associated with extrathyroidal extension. Furthermore, the overexpression of androgen receptor in thyroid cancer cell lines leads to an attenuation of the tumoral phenotype of the cell lines, reducing cell migration and modulating some markers of epithelial to mesenchymal transition [165]. Considering the data on androgen receptor under-expression and its role in tumor aggressiveness, it is surprising that the male gender is associated with a more severe outcome [157,158].

The better prognosis of thyroid cancer in women is not permanent throughout life. Indeed, the recurrence risk of women over 50–55 years of age is similar to the risk of male patients, and the apparent protecting effect of the female sex disappears around the age of menopause [157,158,159]. The expression of ERα has been reported both to be lower [166] and higher [93] in thyroid cancer in post-menopausal women compared to younger patients. The ERα may thus be regarded both as a promoting factor for tumoral growth and as an attenuating factor for the aggressiveness of the tumor. However, the role a change of its expression around the age of menopause may play in the thyroid cancer outcome, is not clearly demonstrated.

After correction for later diagnosis and subsequent more advanced stages of cancer in men, it is logical to consider that lower estrogen or higher androgen concentrations would be the explanation, together with different respective receptor expression, for poorer outcome in men. This gender difference in prognosis is true early in life, which should attenuate the impact of different behavior in access to medical care. The higher frequency of thyroid cancer is visible already before the age of 20, starting with puberty, and the male gender already constitutes a risk factor for recurrence or mortality [167]. Another hormone may be suspected to account for the sex difference in the thyroid cancer prognosis. Indeed INSL3 (Insulin-like 3), the hormone responsible for the testicular descent during embryogenesis and a marker of leydig cell function, circulates at much higher concentration in men than in women [168]. Its receptor, RXFP2, has been described on thyroid cancer cells, and a growth promoting effect of INSL3 on thyroid cells has been shown [169,170]. However, as INSL3 has been shown to be produced in the thyroid tumors themselves, a paracrine rather than endocrine effect may be suspected, which would limit the impact of gender. The presence of functional RXFP2 on thyroid cells may be cell line-dependent, and/or may involve unusual signaling pathways as we were not able to demonstrate INSL3 induced cAMP increase (Suteau V unpublished). In addition, although higher in men than in women, the INSL3 circulating concentration is far lower than the concentration shown to be active on thyroid cells in vitro [169,170].

## 4. Is the Response to Treatment also Gender Biased?

Looking carefully at large studies, and scrutinizing prognosis factors, the worse prognosis in men seems to be an independent factor that does not relate to a poorer response to therapy. There is no indication that refractoriness to I131 treatment would be more frequent in men.

In addition, although the worse prognosis in men appears to be limited to the population with a tumoral BRAF mutation [161], the different clinical trial assessing the capacity of targeted therapies (anti-growth factor, tyrosine kinase inhibitors, MAP kinase inhibitors) to control the progression of metastatic thyroid cancer do not report a sex bias in the response rate.

Although there has been a limited number of immunotherapy trials in thyroid cancer, there is no indication of a differential response rate between women and men. This may appear surprising, considering the higher frequency of thyroid autoimmune diseases in women [1]. However, there is no sex difference in the frequency of thyroid injury induced by targeted therapy or immunotherapy [171,172,173].

## 5. In Conclusion

The frequency of clinically relevant thyroid cancer appears to be really higher in women than men and is related to the life time of reproductive activity, even after correction for bias in behavior and access to medical care.

Estrogens have a positive effect on proliferation and may contribute to mechanisms leading to DNA damages, thus participating in the initiation of tumorogenesis.

The better prognosis in women, after correction for later diagnosis in men, is limited also to the time of reproductive activity. This makes it probable, or at least produces a valid hypothesis, that there is a role played by estrogens or other factors associated with the female fertile period duration in both the promotion and limitation of the oncogenic process.

The occurrence of microcarcinomas, may depend on mechanisms equally present and active in males and females, whereas subsequent events, leading to the growth of the microcarcinoma, may be more frequent in females.

## Data Availability

Not applicable.
